# First person – Tushna Kapoor and Pankaj Dubey

**DOI:** 10.1242/bio.041756

**Published:** 2019-02-15

## Abstract

First Person is a series of interviews with the first authors of a selection of papers published in Biology Open, helping early-career researchers promote themselves alongside their papers. Pankaj Dubey and Tushna Kapoor are co-first authors on ‘Atypical septate junctions maintain the somatic enclosure around maturing spermatids and prevent premature sperm release in *Drosophila* testis’, published in BiO. Pankaj is a Postdoctoral fellow in the lab of Dr Subhojit Roy at Wisconsin Institute of Medical Research (WIMR) II, Madison, USA, investigating how to understand how cytoskeleton dynamics shapes neuronal physiology and transport. Tushna is a PhD student in the lab of Prof. Krishanu Ray at the Tata Institute of Fundamental Research, Mumbai, India, investigating the interplay between cell adhesion, the cytoskeleton and membrane dynamics.


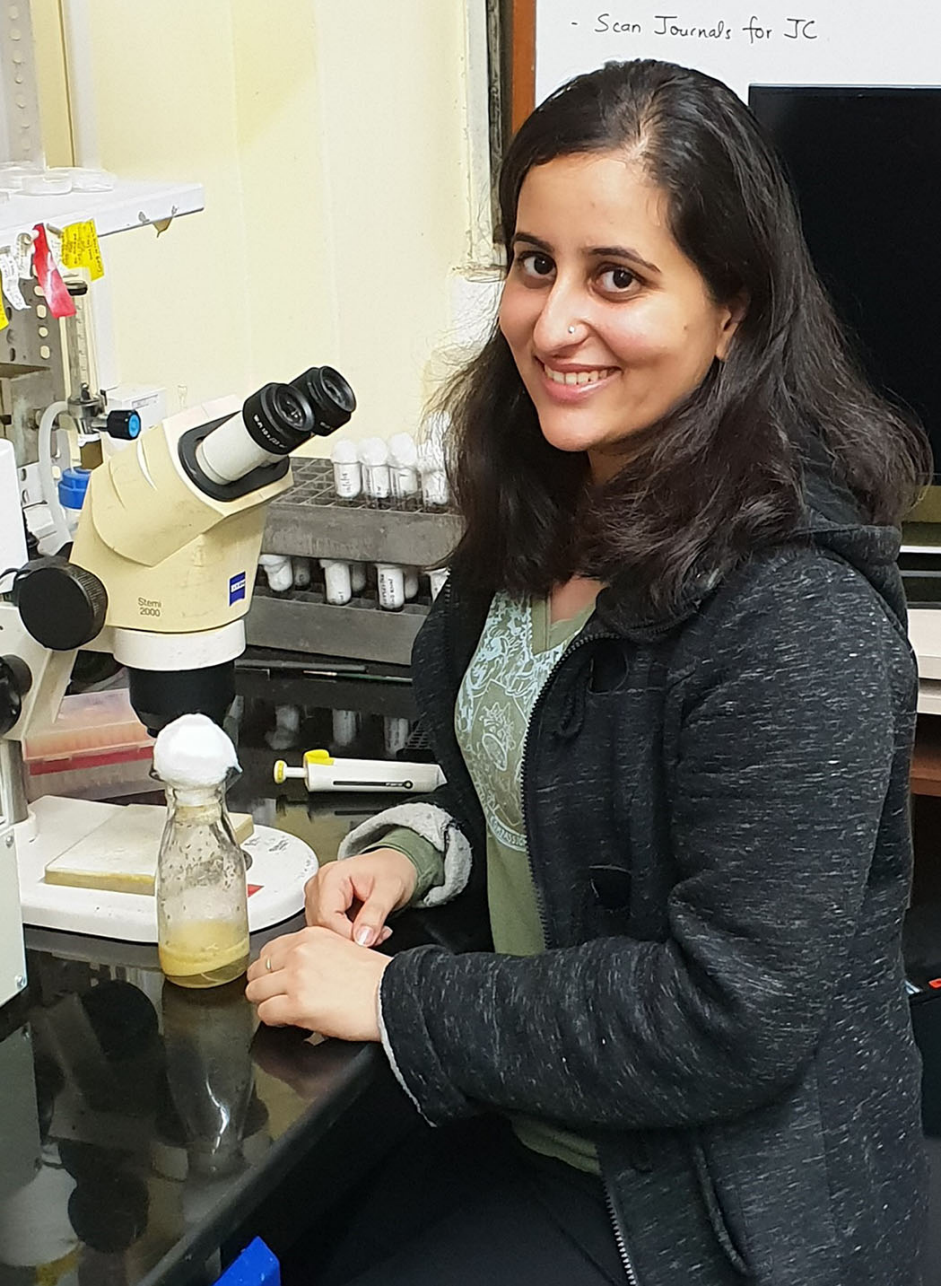


**Tushna Kapoor**

**What is your scientific background and the general focus of your lab?**

**TK-** I completed my BSc (Honours) in Biochemistry from Sri Venkateswara College, University of Delhi, and then joined the integrated MSc-PhD program at the Tata Institute of Fundamental Research (TIFR). My research training at TIFR has been in core cell biology and microscopy.

Our lab focuses on the cellular biology of the cytoskeleton, motors and transport in *Drosophila*. We explore the role of Kinesin-2 in development and function of cilia and axonal transport. A few years back, we stumbled upon the role of Kinesin-2 in maturation of sperm. Since then, the lab has also been studying the role of the cytoskeleton, adhesion and signaling in *Drosophila* spermatogenesis. We employ genetic tools, biochemistry and imaging to understand the cell biology of each project.


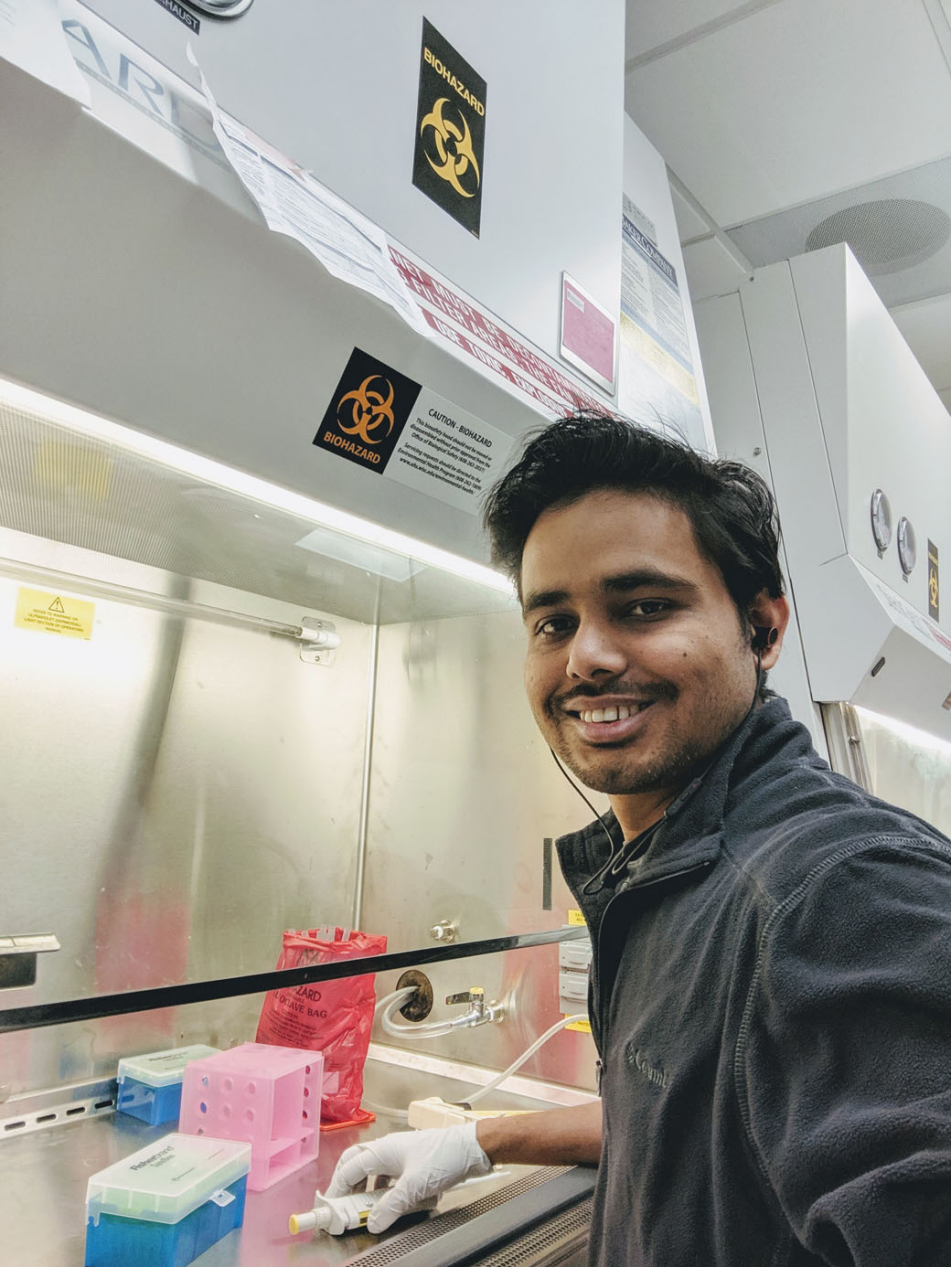


**Pankaj Dubey**

**How would you explain the main findings of your paper to non-scientific family and friends?**

**PD**- This is bit of a tricky one; the non-scientific community always find it amusing that we work on insect sperm release. I explain to them that we are looking at how two cells imprison sperm and we found the ‘lock’ of this prison. When we break the lock, sperm are released early, affecting the ability of insects to produce more insects. It makes an interesting prison-break story.

**TK-** I tell them that a group of sperm cells develop within an enclosure formed by two covering nurse cells. This specialized environment is essential for sperm development. Thus, this enclosure needs to be maintained as the sperms divide, elongate to form a tail and subsequently mature. Therefore, a ‘glue-like’ or ‘sticking’ protein is required for the two covering cells to stick with each other and maintain the enclosure through all these developmental processes. The work in this paper describes the maturation and role of these proteins (septate junctions) through different stages of sperm development.

“The non-scientific community always find it amusing that we work on insect sperm release […] it makes an interesting prison-break story.”

**What are the potential implications of these results for your field of research?**

**PD-** It was believed that two somatic cyst cells are attached to each other throughout spermatogenesis. Our data systematically provide evidence of the existence and maturation of junctions that may keep them together throughout spermatogenesis. We explored the hypothetical implication of destroying these junctions at the final stages of spermatogenesis. Our data will compel the field to study the role of these junctions during the middle stages of spermatogenesis and exploration of more such structures. Although analog structures – tight junctions – are present during mammalian spermatogenesis, the kind of cell biological insight we can get using *Drosophila* is somewhat difficult in mammals. We hope our results further the cause of the essential role of cell adhesion in various developmental contexts.

**TK-** *Drosophila* has been an underappreciated model system to study spermatogenesis. It is, however, strikingly similar to mammalian spermatogenesis, along with the ease of genetic manipulation and imaging. One such example is presented here with the junctions imparting barrier and signaling functions during the early stages, and a structural role during the release stages.

This paper also illustrates the remodeling, maturation and roles of septate junctions in a system other than a typical epithelial layer, where they are most commonly studied.

**What has surprised you the most while conducting your research?**

**PD-** In general, working with *Drosophila* spermatogenesis, I get fascinated by the way system is so beautifully organized. In contrast to mammals, insect sperms are huge – almost as long as the length of their testis. Hence, the containment of growing spermatids becomes essential for spermatogenesis. Having a cell enclosure stitched by septate junctions makes up an elegant solution to avoid the mess that otherwise would have happened with hundreds of 1.8 mm long spermatids floating in *Drosophila* testis. The septate junctions are not known for extensive changes once they are formed and we were surprised to see the dynamic reorganization of the junctions as the spermatid matured.

**TK-** I experienced first-hand how, even with limited techniques and lack of live imaging, scientists could make accurate observations and predictions on dynamic processes. Previous electron microscopy (EM) work has shown that the mature junctions are assembled only in later stages of spermatogenesis, and it was proposed that the position of this junction moves over time and with the developmental stage. We were able to reaffirm these results by live confocal imaging of fluorescently tagged proteins. It was pleasantly surprising.

“I experienced first-hand how, even with limited techniques and lack of live imaging, scientists could make accurate observations and predictions on dynamic processes.”

**Figure:**
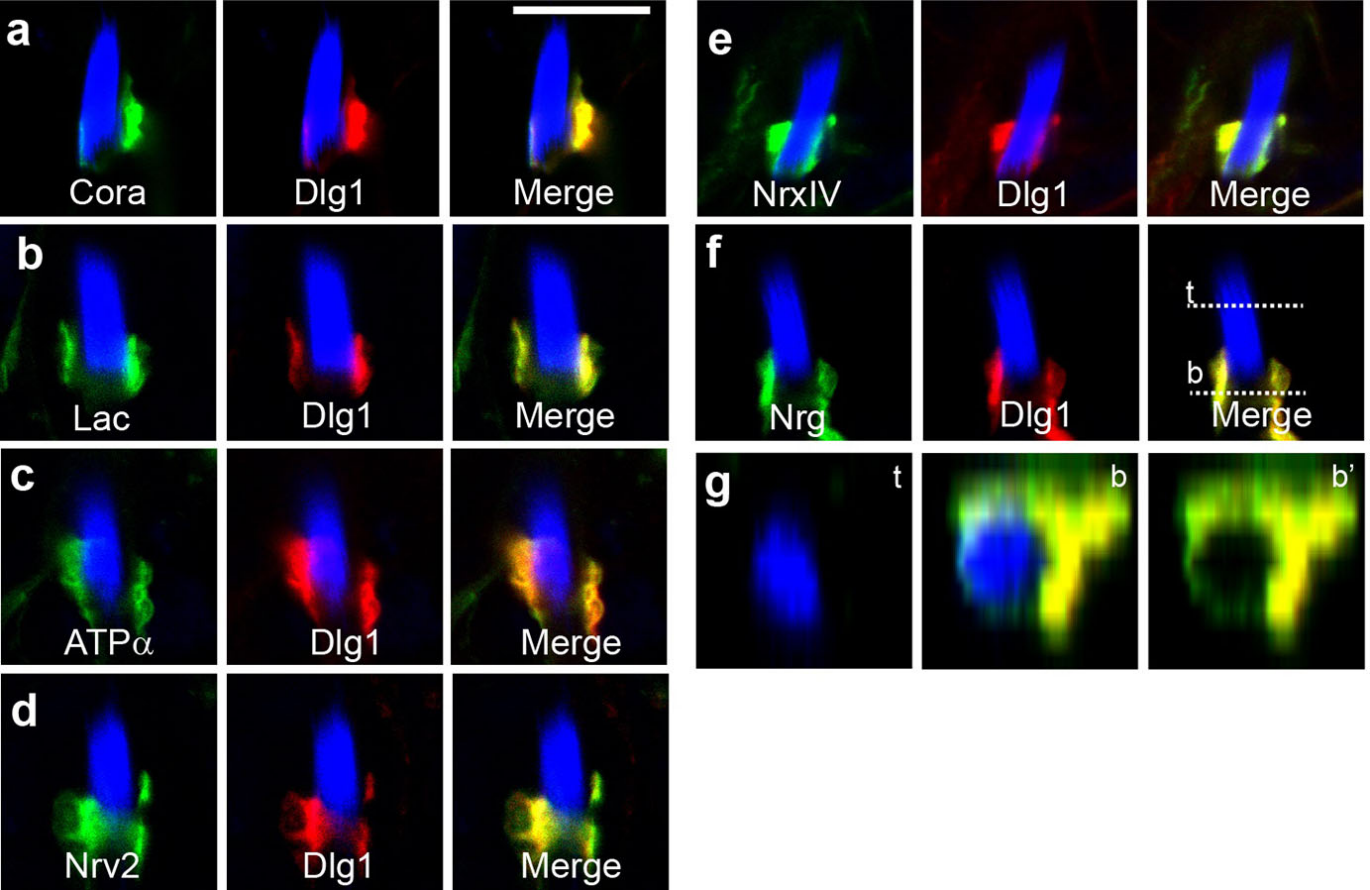
**Bundles of sperm heads stained with Hoescht (blue), which marks DNA, along with the localization patterns of various SJ proteins (green and red).** All tested proteins (green) are co-localized with Dlg (red) between the two somatic cyst cells which enclose the developing sperms. g shows an X-Z cross-section across the nuclei.

**What, in your opinion, are some of the greatest achievements in your field and how has this influenced your research?**

**PD-** There are three important achievements that have driven the spermatogenesis research in *Drosophila*. Along with other great researchers, the late Dr Tokuyasu's meticulous characterization of *Drosophila* spermatogenesis stages in the 1970s using transmission EM was an extraordinary achievement. The six seminal papers from Tokuyasu are essential reads even today for everyone working on *Drosophila* spermatogenesis.

**TK-** I think the collective body of work over many years that show that sites of cell adhesion are not just that, but also hubs of signaling and polarity, have been great findings. Also, recently there have been a flurry of papers that show that, apart from their conventional role of providing a permeability barrier, tight/septate junctions also provide structural and mechanical integrity to cells/tissues. This is exciting as it shows that there is room to explore even in seminal discoveries and that the boundaries are always blurring in science. Both these aspects of septate junctions have been highlighted in our paper as they were shown to have different roles at different stages of spermatogenesis.

**What changes do you think could improve the professional lives of early-career scientists?**

**PD-** I think good mentorship, a cheerful lab atmosphere and space to make mistakes is key to start developing independent thinking. I think is it also important to remind early scientists to keep their personal lives in focus while getting immersed in experiments.

**TK-** Funding is a crucial factor. Both at the level of research, as well as pay-scales of early-career scientists. Many people drop out of science because of the poor pay versus the physical, emotional and mental effort that goes into this career. Also, I think the scientific community needs to inculcate a greater respect for people's lives outside the lab ­– be it their personal or family lives.

“I think good mentorship, a cheerful lab atmosphere and space to make mistakes is key to start developing independent thinking.”

**What's next for you?**

**TK-** I am currently working on another paper where we show the role of an actin-based structure in preventing abnormal sperm release. Apart from that, I also have another project. I plan to complete my PhD within the next 18 months and then move on to do a post-doc within the fields of cell adhesion-cytoskeleton-membrane dynamics.
